# Diagnosis

**DOI:** 10.1007/s11606-026-10342-5

**Published:** 2026-03-23

**Authors:** William M. Tierney

**Affiliations:** Department of Community and Global Health, Richard M. Fairbanks School of Public Health, Indiana University, Indianapolis, IN USA

I got the news — my memory’s lacking

I've got the blues — cognition’s cracking

I feel the dread of fraying threads of time

But thoughts aren't clogged, my head’s not fogged, life’s still sublime!

Dementia’s sword — Damocles swinging

My friends are floored — yet birds keep singing

I'm still alive and strive to drive my brain

Can hopes still sail through darkening veils of misty rain?

So what’s next now — I stop to ponder

I don’t know how — to plan or wander?

I feel the same yet won't remain right here

I’ll fight decline and won’t resign to gnawing fear

I'm not alone — my loved ones gather

They give me strength — my story matters

When the sun is gone then I’ll become the moon!

My love abides while neurons die all far too soon

